# Normative values of quantitative sensory testing in Hispanic Latino population

**DOI:** 10.1002/brb3.466

**Published:** 2016-05-17

**Authors:** Alejandra González‐Duarte, Mónica Lem‐Carrillo, Lorena Guerrero‐Torres

**Affiliations:** ^1^ Department of NeurologyInstituto Nacional de Ciencias Médicas y Nutrición Salvador ZubiránMéxico CityMéxico

**Keywords:** Hispanic, quantitative sensory, testing, thermal thresholds, hispanic

## Abstract

**Background:**

Quantitative Sensory Testing (QST) is more often used because of the increasing recognition of small fiber neuropathy.

**Methods:**

We studied QST in a systematic way in an age‐stratified cohort of 83 neurological‐free Hispanic Latinamerican patients. Predefined standardized stimuli were applied using the method of limits.

**Results:**

WDT range from 2.2 to 3.3°C in hands, and from 4.0°C up to 6.6°C in feet. Cold detection threshold range from 2.2 to 3.6°C in hands, and from 2.6°C to 4.5°C in feet. Heat‐induced pain (HP) was induced at lower temperatures than previously reported, with a range from 41.8°C to 44.5°C in hands and from 43.2 to 45.7°C in feet. Similar to HP, cold pain was also induced at much higher temperatures, between 21.4–17.3°C in hands and 21.5–16.5°C in feet. Vibratory stimuli ranged from 0.8 to 1.7 *μ*/sec in hands and from 1.4 to 3.5 *μ*/sec in feet.

**Conclusion:**

Temperature and vibration thresholds were similar to those previously reported in other populations except for pain thresholds that were lower in this population than in the Caucasian population.

## Background

Quantitative sensory testing (QST) is being utilized more often because of the increasing recognition of small fiber neuropathy. Controlled clinical and epidemiological trials that monitor peripheral nerve functions are introducing QST to the array of studies. Also, because QST can quantify sensory deficits easily, it is beginning to be used regularly in the clinical practice. Although conventional sensory nerve conduction studies evaluate large nerve fibers function, QST examines the function of small A‐delta and C nerve fibers, including the corresponding central pathways (Krumova et al. 2012; Backonja et al. 2013). It is a psychophysical method used to quantify somatosensory function in response to controlled stimuli. Perception of the thermal thresholds is the most common modality used.

The availability of automated systems, standardization of the instructions to subjects, definition of stimulus characteristics, and similar testing algorithms are features that reinforce its use and decrease the source of error. In spite of this, different sets of normal values are used in different institutions, which have made difficult the interpretation of the test. This can be resolved by deriving each laboratory′s own set of normal values, however, this will be less reliable or reproducible when comparing with other populations.

Pain sensitivity varies substantially among humans (Diatchenko et al. 2005; Robinson‐Papp et al. 2009). Of particular importance is the impact of demographic factors in the sensory perception. Ethnic disparities have been described in thermal and mechanical somatosensory profiles in QST between Chinese and Danes (Yang et al. 2013) or Japanese and Caucasian (Komiyama et al. 2009), A potential explanation of the ethnic disparities is the difference between haplotypes (Diatchenko et al. 2005; Edwards et al. 2005).

QST normal values have been described in two different populations, with varying results between cohorts. In this manuscript, we are reporting the thermal and vibratory thresholds of a Mexican Latin American Hispanic population without neuropathy using similar equipment, methodology, and algorithms than those described previously.

## Methods

We randomly selected 84 healthy subjects between 20 and 70 years old who came to our Institution to visit a patient. Thirty‐four patients were excluded because of suspicion of neuropathy. Before testing, all subjects answered a questionnaire of their medical history, medications and a brief neurological symptom‐screening test. Subjects were eligible when they were born in Mexico from Mexican parents; the clinical history showed no past or ongoing medical disease; they were not taking medications; they did not have a history of consuming potential neurotoxic medications, and did not display any neurological sign or symptom. All subjects gave their written consent to participate in the study in a previously approved ICF by the IRB of our Institution. Instructions were given in the same way by reading them to the patient. Two evaluators who had similar training performed the studies. All studies were executed in the autonomic laboratory that has controlled room temperature and humidity and is sound isolated. Room temperature was maintained at 21–24°C and skin temperature at 32°C throughout testing. Cellular phones were turned off during the study. Patients with obvious signs of anxiety or who did not understand fully the instructions were eliminated. Results were analyzed according to five different groups of age, from 20 to 30, 31 to 40, 41 to 50, 51 to 60, and 61 to 70 years old.

A TSA‐II neurosensory analyzer (Medoc^**®**^, Ramat Yishai, Israel) was used with the standard 30 × 30 mm thermode. Stimuli were applied to the thenar region on the left hand and dorsal surface on the left foot. The magnitude of the stimuli was previously standardized with the method of limits (METHOD LIMITS) as a ramped ascending or descending stimuli (1°C/sec); the operator always read the same instructions in Spanish before the beginning of the test. Cold and warm detection thresholds (WDTs) were measured first (CDT, WDT) and then cold pain and heat pain were determined (CP, HP). Subjects indicated when the stimulus was initially felt or painful and the operator stopped the stimuli. The mean threshold temperature of three consecutive measurements was calculated. The baseline temperature was 32°C with cut‐off temperatures form 0 to 50°C. Vibratory stimulus was then applied in the third finger and first toe.

### Statistical methods

Samples were divided in five groups, according to age. The media of each threshold were obtained. Two‐way Students t tests were used to compare groups. All data were normally distributed in log‐space. All calculations were performed using SPSS version 20 (IBM). All data are presented as mean and SD.

## Results

Fifty subjects were analyzed from age 21 to 70 years old (Table 1). The mean age was 44.6 ± 1.48 years, 21(42%) were men, and 29(58%) were women. Mean body mass index was 25.3 ± 0.4, very similar in each group. All patients were Hispanic and Latino, born in Mexico from two Mexican parents. Healthy subjects were identified according to their medical history.

**Table 1:** Demographics

Group of age (*n* = 50)	Mean age/SD	Gender, male (%)	BMI
21–30 years old (*n* = 10)	26 ± 0.6	8 (50)	24 ± 0.9
31–40 years old (*n* = 10)	34.76 ± 0.86	11 (65)	25.1 ± 0.96
41–50 years old (*n* = 10)	45.5 ± 0.63	6 (30)	26.9 ± 0.82
51–60 years old (*n* = 10)	55.13 ± 0.45	2 (13)	26.06 ± 0.78
61–70 years old (*n* = 10)	64 ± 0.55	2 (13)	24.6 ± 0.64

BMI, Body mass index.

The thermal sensory testing element measures the thresholds for four sensory submodalities, WDT, cold detection threshold (CDT), heat‐induced pain (HP), and cold‐induced pain (CP). WDT is mediated by C fibers and it is usually one or 2°C above the adaptation temperature of 32°C (Dyck et al. 1993; Perkins and Bril 2003; Shy et al. 2003). Results are shown in Table 2 and 3. We found a range from 2.2 to 3.3°C in hands (34.2–35.3°C), similar to what has been described previously, but of 4.0°C and up to 6.6°C in feet (36–38.8°C), above the limits described by DFNS (Maier et al. 2010), but in the ranges of the Mayo Clinic study (Dyck et al. 1993). As described previously, usually the most altered thresholds were found in older subjects. Cold detection thresholds are mediated by A‐delta fibers and for normal subjects are usually one or 2°C below the adaptation temperature of 32°C (Yarnitsky et al. 1995; Perkins and Bril 2003; Shy et al. 2003). We found a range from 2.2 to 3.6°C below the adaptation temperature in the hands (29.8–28.4°C), and from 2.6 to 4.5°C in the feet (29.4–27.5°C). Mean values of each different group are described in Table 2.

**Table 2:** Normative values of QST per group of age

Age (yo)	Cold detection threshold (°C)	Δ (°C)	Warm detection threshold (°C)	Δ (°C)	Cold‐induced pain (°C)	Heat‐induced pain (°C)	Vibration threshold (*μ*/sec)
Hand (Left thenar area)							
20–30	29.9	2.1	34.2	2.2	18.3	42.3	0.8
31–40	29.4	2.6	34.2	2.2	21.4	43.2	1.2
41–50	28.4	3.6	35.1	2.4	20.7	42.1	1.0
51–60	28.5	3.5	34.6	2.6	17.3	41.8	1.7
61–70	29.1	2.9	35.3	3.3	20.8	44.5	1.5
Foot (Dorsal surface)							
20–30	29.4	2.6	37.4	5.4	21.5	43.3	1.4
31–40	27.5	4.5	37.2	5.2	19.3	44.1	3.0
41–50	27.8	4.2	36.2	4.0	16.5	42.3	3.4
51–60	28.6	3.4	37.6	5.9	19.8	43.2	3.5
61–70	28.7	3.3	38.6	6.6	17.8	45.7	2.6

yo, years old; QST, Quantitative sensory testing.

**Table 3:** Standar deviations in QST thresholds

Group	SD −3	SD −2	SD −1	MEAN	SD +1	SD +2	SD +3
Cold threshold hand (Left thenar area)							
20–30	26.93	27.92	28.91	29.90	30.89	31.88	32.87
31–40	25.65	26.90	28.15	29.40	30.65	31.90	33.15
41–50	22.44	24.26	26.08	27.90	29.72	31.54	33.36
51–60	22.80	24.70	26.60	28.50	30.40	32.30	34.20
61–70	24.37	25.94	27.52	29.10	27.52	32.26	33.83
Warm threshold hand (Left thenar area)							
20–30	32.89	33.35	33.82	34.29	34.76	35.23	35.69
31–40	31.84	32.62	33.41	34.20	34.99	35.78	36.56
41–50	29.29	31.58	33.86	36.15	38.44	40.72	43.01
51–60	31.00	32.22	33.43	34.64	35.85	37.06	38.28
61–70	27.97	30.41	32.86	35.30	37.74	40.19	42.63
Cold threshold feet (Dorsal surface)							
20–30	30.18	31.55	32.92	34.29	35.66	37.03	38.40
31–40	27.53	29.75	31.98	34.20	36.42	38.65	40.87
41–50	28.04	30.74	33.45	36.15	38.85	41.56	44.26
51–60	28.56	30.59	32.61	34.64	36.67	38.69	40.72
61–70	30.91	32.38	33.84	35.30	36.76	38.22	39.69
Warm threshold feed (Dorsal surface)							
20–30	26.21	28.90	31.60	34.29	36.98	39.68	42.37
31–40	27.81	29.94	32.07	34.20	36.33	38.46	40.59
41–50	32.59	33.78	34.96	36.15	37.34	38.52	39.71
51–60	25.89	28.81	31.72	34.64	37.56	40.47	43.39
61–70	23.06	27.14	31.22	35.30	39.38	27.14	23.06

QST, Quantitative sensory testing.

Heat‐induced pain (HP) is mostly mediated by C fibers with some involvement of A‐delta fibers. Thresholds are usually around 45°C (Dyck et al. 1993; Yarnitsky et al. 1995). We found that pain was induced in our population at lower temperatures, with a range from 41.8 to 44.5°C in hands and from 43.2 to 45.7°C in feet. Cold‐induced pain (CP) is mediated by a combination of both C and A‐delta fiber. It is the most variable and difficult to assess of all previous modalities, at about 10°C (Dyck et al. 1993). Similar to HP, CP was also induced at much higher temperatures than the ones described in other populations, between 21.4 to 17.3°C in hands and of 21.5 to 16.5°C in feet. Comparison between populations is shown in Table 4.

**Table 4:** QST comparison between the German Network Protocol, Mayo Clinic, and Mexican values

	DFNS (*n* = 180)	Dyck (*n* = 7)	Mex (*n* = 50)	Mean	95% CI	*P*
CDT (° from baseline= Δ)						
Hand	−1.2°C	−3.2°C	−2.9°C	−2.4	0.2 to 5.0	0.05
Feet	−2.2°C	−5.1°C	−3.6°C	−3.7	0.0 to 7.2	0.04
WDT (° from baseline= Δ)						
Hand	1.8°C	6.9°C	2.5°C	3.7	−3.13 to 10.6	0.49
Feet	4.1°C	9.7°C	5.4°C	6.4	−0.8 to 13.6	0.63
CP						
Hand	13.2°C	21.8°C	19.7°C	17.5	6.8 to 28.2	0.01
Feet	12.6°C	20.8°C	18.9°C	17.4	6.7 to 28.0	0.02
HP						
Hand	43.3°C	46.8°C	42.7°C	44.2	38.7 to 49.7	0.00
Feet	44.4°C	44.7°C	43.7°C	44.2	42.9 to 45.5	0.00
Vibration						
Hand	0.9* μ*/sec	–	1.2* μ*/sec	0.85	0.21 to 1.4	0.03
Feet	0.8* μ*/sec	–	2.8* μ*/sec	2.0	−8.1 to 12.1	0.24

WDT, warm detection threshold; CDT, cold detection threshold; HP, heat pain; CP, cold pain; DFNS, German Research Network on Neuropathic Pain; QST, quantitative sensory testing.

Values were taken from 1.5°/sec with the large (10 cm^2^) thermode.

Non‐nociceptive cool stimuli are mediated by A‐delta small myelinated fibers, whereas warm stimuli and nociceptive stimuli are mediated by C fibers. Vibration thresholds can also be assessed, which will reflect the function larger A*β* fibers.

The computerized device measures thresholds for vibratory stimuli at the range from 0 to 130 μm and at the rate of 0.1 to 4.0 μm/sec with wide variations between populations. We found the same difference between upper and lower extremities than with the other submodalities, where thresholds were higher in the lower extremities. Values ranged from 0.8 to 1.7 *μ*/sec in hands and 1.4 to 3.5 *μ*/sec in feet.

## Discussion

Research studies have confirmed the utility of QST for the assessment and monitoring of somatosensory deficits, particularly in diabetic and small fiber neuropathies. When appropriate standards are applied, QST can provide important and unique information about the functional status of somatosensory system, which would be complementary to already existing clinical methods. However, thermal detection reference values from different studies have been inhomogeneous (Dyck et al. 1993; Yarnitsky et al. 1995; Perkins and Bril 2003; Shy et al. 2003; Maier et al. 2010) in 1993 published the first reference values of QST in normal controls using the Computer‐assisted sensory examination (CASE IV) system and comparing different methods of testing. Later, the German Network Study (DFNS) (Rolke et al. 2006; Maier et al. 2010) performed the largest database of healthy subjects using the Medoc system. Reference values in this study were taken from a multicenter study, and differences between centers were observed. To our knowledge, normal values for the Latin American Hispanic population have never been reported as a single group before.

Our methods were similar to those reported earlier, however, because stratification into two age groups (young < 40 years; old < 40 years) as the DFNS database (Maier et al. 2010) and in other publications may lead to age‐related bias if used diagnostically, we used a different age stratification system by age decades, similar to the redefined stratification by Magerl et al. (2010). Also, the technological system we used was similar to the DFNS but different to the study performed by Dyck et al. (1993). Despite those differences, we showed very similar values in WDT, HP, and VT between populations. However, cold‐induced pain thresholds were considerably lower than the DFNS, but similar to what was described in Dyck′s et al. original cohort. Cold hyperalgesia is an important positive sensory sign for non‐nociceptive parameters, particularly, in patients with complex regional pain syndrome, trigeminal neuralgia, and herpes zoster. It has been regarded as less specific due to the fact that has been considered with large variability in healthy subjects Maier et al. (2010). However, cold‐induced thresholds in this study were very similar to Dyck′s study, around 20°C.

Correctness of result interpretation using the DFNS reference data is difficult, therefore we provided an over simplistic analysis to provide reference values in the Hispanic population.

Thermal sensory testing proved to be similar to other studies, except for temperature induced‐pain thresholds. This has previously been inferred in clinical trials where pain thresholds had been different in other Hispanic cohorts (Diatchenko et al. 2005; Robinson‐Papp et al. 2009). Small differences were also noted between hands and feet, being usually higher thresholds in the upper extremities. Similarly, older age controls had higher and broader thresholds, but unfortunately there were no statistical significant differences among age‐stratified groups, probably because of the small sample size. However, there is a trend toward the higher thresholds in older subjects and it is consistent with what is seen in the clinical practice and also described elsewhere (Huang et al. 2010). Limitations of this study include the lack of separation between genders. It is possible that performing a complete neurological exploration and obtaining laboratory studies could have unmasked subclinical neuropathy in some subjects, however, this intended to be a real‐life population study. Also, different methods and different systems were used in the two studies we compared our results to. However, we think that this set of values in a Hispanic population will provide useful information to clinicians and researchers when using QST.

## Disclosures

This manuscript did not receive financial aid. The authors of this manuscript have nothing to disclose.

## Conflict of Interest

None declared.
